# A Third Approach to Gene Prediction Suggests Thousands of Additional Human Transcribed Regions

**DOI:** 10.1371/journal.pcbi.0020018

**Published:** 2006-03-17

**Authors:** Gustavo Glusman, Shizhen Qin, M. Raafat El-Gewely, Andrew F Siegel, Jared C Roach, Leroy Hood, Arian F. A Smit

**Affiliations:** 1 Institute for Systems Biology, Seattle, Washington, United States of America; 2 Institute of Medical Biology, University of Tromsø, Tromsø, Norway; 3 Departments of Management Science, Finance and Statistics, University of Washington, Seattle, Washington, United States of America; University of California Santa Cruz, United States of America

## Abstract

The identification and characterization of the complete ensemble of genes is a main goal of deciphering the digital information stored in the human genome. Many algorithms for computational gene prediction have been described, ultimately derived from two basic concepts: (1) modeling gene structure and (2) recognizing sequence similarity. Successful hybrid methods combining these two concepts have also been developed. We present a third orthogonal approach to gene prediction, based on detecting the genomic signatures of transcription, accumulated over evolutionary time. We discuss four algorithms based on this third concept: Greens and CHOWDER, which quantify mutational strand biases caused by transcription-coupled DNA repair, and ROAST and PASTA, which are based on strand-specific selection against polyadenylation signals. We combined these algorithms into an integrated method called FEAST, which we used to predict the location and orientation of thousands of putative transcription units not overlapping known genes. Many of the newly predicted transcriptional units do not appear to code for proteins. The new algorithms are particularly apt at detecting genes with long introns and lacking sequence conservation. They therefore complement existing gene prediction methods and will help identify functional transcripts within many apparent “genomic deserts.”

## Synopsis

To date, genes have been identified from genomic sequence using two basic concepts: the identification of specific signals delineating the structure of the genes and by similarity to previously known genes. Here the authors describe four novel algorithms based on a third basic concept: the identification and quantification of mutational and selectional effects of transcription. Central to this work is a detailed analysis of interspersed repeats, the “junk DNA” left behind by transposon activity, that is usually discarded when predicting genes even though it amounts to nearly half the human genome. Using the new methodology, the authors identify thousands of potential novel genes, some of which appear not to code for protein products. The new algorithms are particularly apt at detecting genes with long introns and lacking sequence conservation. They therefore complement existing gene prediction methods and will help identify functional transcripts within many “genomic deserts,” regions currently thought to be devoid of genes.

## Introduction

The current annotation of human genes is likely to be incomplete, particularly for genes not coding for proteins. Wong et al. [1] have argued that the vast majority of the human genome is transcribed. Indeed, there is mounting experimental evidence showing that the human transcriptome is more complex and extensive than previously thought (Cheng et al. [2] and references therein). Computational sequence analysis can help direct the experimental discovery of novel genes; even the highly experimentally annotated Caenorhabditis elegans ORFeome was significantly enriched by computational gene predictions [3].

Several types of software for gene prediction are currently available. There are two basic concepts underlying these methods: (1) the recognition of gene structure and (2) sequence similarity. Methods that predict gene structure identify the structural and functional requirements for a segment of genomic sequence to be able to be transcribed, spliced, and finally to be translated into a protein sequence, e.g., GenScan [4]. Methods based on sequence similarity identify genes by detecting regions of sequence that have been conserved in evolution, e.g., spliced alignment in Procrustes [5]. The two concepts have been combined leading to significant improvements in accuracy [6,7]. Finally, gene predictions can be validated computationally by comparison to gene structures derived from coalignment and clustering of locus-specific mRNAs and ESTs on the genomic sequence.

Here, we develop a third basic concept in gene prediction that is based solely on the analysis of genomic sequence data: the recognition of “transcription footprints.” These are the side effects of sustained transcription on the genomic sequence, leading over evolutionary time to an accumulation of differences between the two DNA strands, which can be detected by appropriate statistical analysis. We present here an integrated suite of four prediction methods exemplifying this third basic concept. Importantly, the method presented here can readily predict transcriptional units that do not code for proteins.

Many of the transcription footprints are buried in interspersed repeats, which comprise almost half of the human genomic sequence, including intronic sequences. Most of these repeats are copies of transposable elements exhibiting various levels of sequence decay and are systematically excluded (“masked”) as a first step in most standard gene prediction methodologies. The present work takes advantage of a detailed analysis of interspersed repeats, as a reference framework for detecting the otherwise overlooked transcription footprints.

## Results

### Strand Biases as Transcription Footprints

In this work, we focus on the detection of transcription footprints in the form of significant differences between the “forward” (coding, sense) and “reverse” (antisense) strands of transcribed regions. Two sources for orientation biases within genes are (1) mutations influenced by the act of transcription and (2) selection against harmful signals that disrupt transcription.

#### Biased mutation.

A transcription-associated strand asymmetry has been described [8] and attributed to a byproduct of transcription-coupled DNA repair in germline cells. Transcription encourages the early resolution of mutations from polymerase base misinsertions, which are biased toward purines [9]. Two gene prediction algorithms taking into account these biases were suggested [8]: a “nucleotide compositional analysis,” which identifies composition skews in the sequence studied, and a “substitution rate analysis,” identifying all lineage-specific mutations from multispecies alignments.

#### Biased selection.

The identification of ORFs has long been considered the method of choice for identifying genes in genomes with little or no splicing. Where mRNA splicing is prevalent, this method loses power since ORFs can be split into several short segments separated by potentially long introns. In conceptual similarity to ORF identification, we hypothesized the existence of selection against the introduction of any signal that could prematurely interrupt the transcription process, in particular, polyadenylation signals (PASs). Since the PAS is asymmetric, with consensus sequence AATAAA or ATTAAA, selection should lead to orientation biases: introduction of the same signal in the opposite orientation would typically be a neutral event.

### Four Novel Transcript Predictors

**Figure 1:**
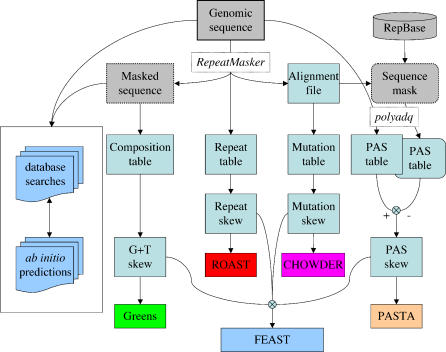
Information Flow in FEAST The genomic sequence is analyzed using RepeatMasker, yielding a masked sequence (studied for its base composition), a repeat table, and an alignment file, which is used to list mutations in repeats and to produce a “sequence mask.” Both the original sequence and the sequence mask are studied using polyadq, yielding tables of predicted PASs. The nucleotide composition of the unique sequence, and the mutations within repeats, is tabulated as well. The tables are then analyzed to calculate skews, which are finally used to produce predictive scores, separately for each method (Greens, ROAST, CHOWDER, and PASTA) or in combination (FEAST).

We describe here four algorithms for transcript prediction and then present their combination as an integrated method (Figure 1).

#### The first algorithm: Greens.

The combined mutational biases yield an excess of G + T over A + C in the forward strand of genes, leading to an equilibrium value of 52.7% G + T [8,10]. The nucleotide compositional analysis employs a log-likelihood ratio system derived from this expected nucleotide composition at equilibrium and then identifies segments of sequence with significant orientation biases. In this method, though, the strongest scores are obtained by the most biased sequences, not those nearest the equilibrium value (Figure S1A). By training on transcribed genomic sequence, we obtained an empirical distribution describing the strength of strand bias as a function of the local G + T composition (Figure S1B). We found extremely biased nucleotide compositions to be less orientationally biased within genes than the more prevalent moderate compositions. The log-likelihood ratio scores obtained empirically from this analysis represent a better model for saturation of the mutation process and serve as the basis for this first algorithm (Greens), which is a refined version of the published nucleotide composition analysis.

#### The second algorithm: CHOWDER.

The Greens algorithm is not applicable to interspersed repeats, most of which have inserted into the genome too recently to have reached equilibrium with respect to G + T composition [8]; some repeats introduce large compositional distortions, e.g., LINE1 retrotransposons have a 2:1 ratio of A to T in the forward strand [11]. Excluding repeats from the analysis is, though, equivalent to discarding almost half of the genomic data [12]. The alignment of an interspersed repeat copy to its repeat family consensus sequence provides an enumeration of the substitutions it accrued since the time it inserted into the genome (Figure S2A). These substitutions have well-defined evolutionary directionality, from the state observed in the consensus to that observed in the extant sequence. A strand orientation can be established by arbitrarily normalizing mutations to one strand, e.g., the C→T mutation is equivalent to a G→A mutation on the opposite strand. We analyzed the alignments of all repeats contained within known transcripts and tabulated the observed frequencies of the different mutations (Figure S2B). Using log-likelihood ratios derived from these observed frequencies, we implemented this second algorithm for transcript prediction (CHOWDER [CHanges Oriented Within DispErsed Repeats]). As observed for unique sequences, the overall trend for repeats leads to a rise in the frequency of G + T in the forward strand of transcripts. Interestingly, the strongest skews are for the G→T mutation (C→A on the opposite strand), which lowers the G + C content of transcribed regions.

#### The third algorithm: ROAST.

Many retrotransposons have a highly conserved PAS; this suggests that the retroposition of such elements into the genome could be harmful when the orientation of the repeat is the same as the host gene (Figure S3A). We found interspersed repeats of most types to be significantly biased in their orientation within transcribed regions, typically with a preference for the antisense strand [13,14]. We observed a strong correlation between the repeat orientation biases in the human and mouse genomes (Figure S3B). There is also a qualitative correlation between the extent of the orientation bias of a repeat family and the strength of the PAS it introduces into the genome: LTR elements are the most biased, LINE1 elements are strongly biased but less so, and DNA transposons are the least biased. While the LINE1 consensus sequences do not show canonical PAS, approximately 85% of LINE1 insertions terminate at the 3′ end of the consensus sequence, suggesting that active LINE1 retroposons have reasonably strong PASs [15]. It has been suggested that forward-strand LINE1 repeats attenuate gene expression due to its idiosyncratic A-rich nucleotide composition [16]; in this case, we would expect longer LINE1 inserts to be more strongly skewed than shorter ones. We observed LINE1 skews to be invariant with element length (skew = −0.3), suggesting that the biasing mechanism involves a discrete signal (e.g., the PAS) and not a length-dependent compositional signal. DNA transposons do not replicate via their transcript, and read-through transcription is probably of very little effect on their survival. Being transcribed by RNA polymerase III, Alu repeats have no PAS, yet they are significantly biased in orientation. To elucidate why, we performed a simulation experiment in which the consensus sequence for each repeat was integrated into randomly picked locations in a 4.4-Mb human genomic sequence (GenBank accession number NT_001520) in random orientation and studied the location and quality values for the potential PASs observed in the resulting sequences. In this simulation experiment, we found that the combination of the Alu polyA tail with the integration target site frequently generates a novel PAS (not shown). This third algorithm for transcript prediction (ROAST [Repeat Orientation Analysis Suggesting Transcripts]) is based on the statistical quantification of repeat orientation skews, stratified by repeat family and age and by the regional %GC of the sequence.

#### The fourth algorithm: PASTA.

We further hypothesized that selective pressure to maintain functionally transcribed regions “open” to uninterrupted transcription might act against mutations making a weak forward-strand PAS stronger and in turn might favor mutations weakening cryptic PAS-like sites within transcripts (Figure S4A). We identified PAS-like sequences using the polyadq program [17] and indeed found them to be significantly biased within known transcripts, with a strong preference for the reverse orientation. Most of this bias can be explained by random expectation from a G + T–skewed sequence (see above): since the PAS is A rich, it is expected to be more prevalent in the reverse strand of a T-rich sequence (Figure S4B). We therefore implemented a statistical correction to compensate for these expected biases in PAS orientation, based on training on intergenic sequences. Furthermore, the differential fixation of repeats itself yields a skew in PAS favoring the reverse strand. We therefore generated a “sequence mask” in which each repeat was replaced with the corresponding section of its consensus sequence and corrected PAS counts and strengths within repeats to reflect changes from the consensus. Significant PAS biases remain after these corrections, yielding this fourth independent predictor (PASTA [Polyadenylation Signal Transcript Analysis]).

#### The integrated method: FEAST.

We implemented the four methods using the same conceptual structure, as follows. First, we obtained log-likelihood ratio parameters for each predictor by genomewide training on annotated known genes. Second, we scanned the whole genome and tabulated local frequencies and orientations for each of the predictors. Third, we calculated for each genomic location four scores representing the significance level of the strand bias; these scores indicate each method's support for a claim of transcription at that genomic location. These four scores are then combined, yielding an integrated predictive score (Figure 1). Finally, we integrated these scores into maximal segments [18,19], predicted to correspond to transcribed regions. FEAST (Fast Empirical Algorithms Suggesting Transcripts) scores are calculated as Z scores analytically calculated from the distribution expected from an independent assortment of randomly oriented predictors, under the assumption that the four combined methods are mutually independent under the null hypothesis that a region is not transcribed (with observed correlations due to uniformly higher expected scores within transcribed regions). Positive scores indicate evidence for transcription on the forward strand; negative scores support transcription on the reverse strand, and small values of either sign indicate the absence of significant orientation biases. See Materials and Methods and Supporting Information for a detailed description.

### Reanalysis of Known Genes and Gene Predictions

**Figure 2:**
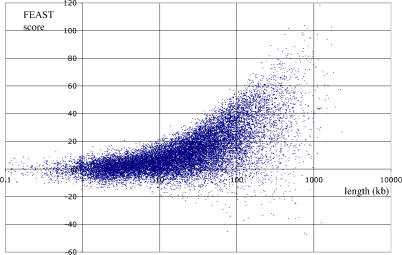
FEAST Reanalysis of Known Genes Scatterplot of FEAST scores versus gene length for known genes from the UCSC Genome Bioinformatics Site [20]. Genes overlapping known genes on the complementary strand were excluded. Scores greater than 3 are considered significant.

**Figure 3:**
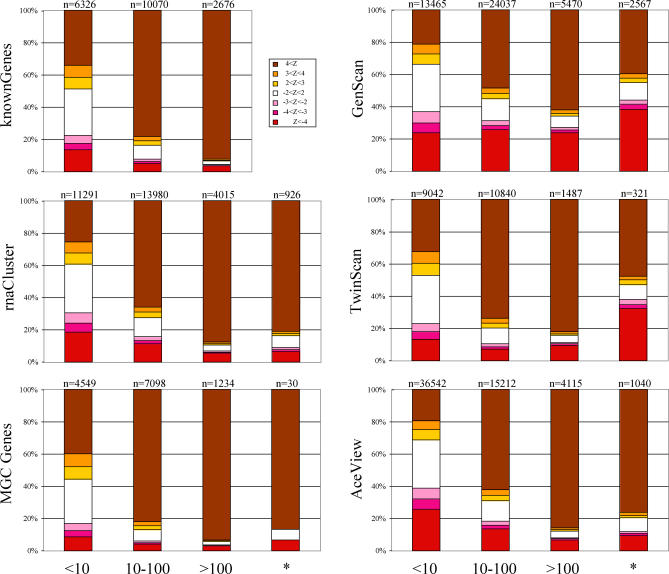
FEAST Reanalysis of Existing Annotation Success rates for FEAST reanalysis of known genes (top left), experimental gene annotations (center and bottom left), and gene predictions (right). Gene annotations were stratified by length into three classes: short (<10 kb), medium (10 to 100 kb), and long (>100 kb); the number of genes in each class is given above each bar. FEAST scores were stratified into nonsignificant (white, −2 < Z < 2), giving significant scores for the expected strand (shades of brown, Z > 2) and giving significant scores for the wrong strand (shades of red, Z < −2). The Z < −4 and Z > 4 bins include potentially large values as displayed in Figure 2. Columns labeled with asterisks include the gene regions longer than 100 kb remaining after subtraction of overlaps with known genes, on which FEAST had been trained.

The FEAST analysis can be used as an independent qualification of known genes and gene predictions, by combining FEAST scores included within the genomic span of each gene. It is important to stress that here we are not comparing two independent sets of transcript predictions but rather calculating FEAST scores for transcribed regions as predicted by other methods, and predicting just the transcript orientation based on our log-likelihood model. We performed this calculation for all known genes in the human genome, excluding those for which an overlapping antisense transcript has been annotated (Figure 2). This analysis shows that the FEAST method typically requires more than 10 kb of sequence to identify the transcript's orientation: a significant positive score (Z > 2) was obtained for only 8% of the genes shorter than 10 kb, rising to 86% for genes longer than 100 kb (Figure 3, top left). Conversely, incorrect orientation identifications (Z < −2) were made for 1.4% of genes shorter than 10 kb but for only 3% of genes longer than 100 kb. Therefore, a useful property of FEAST is that longer transcribed regions typically produce higher scores, making this approach useful for identification of large genes with long introns. Such genes are particularly difficult to identify using current computational gene prediction methodologies.

We observed some extremely negative scores for some annotated genes, typically indicating errors in the annotation. For example, the AF118089 transcript spanning the range chr1:88,617,147–88,738,864 (q→p strand) was assigned a FEAST score of −37.4. This transcript in fact corresponds to the reverse strand of the first nine exons of the *PRKCL2* gene and likely represents a clerical error. Some of the observed negative scores may indicate actual transcription on the reverse strand of those genes.

We similarly calculated FEAST scores for all GenScan, Twinscan, and AceView annotations in the human genome and found them to be largely in agreement (Figure 3, right) while preserving the trend of higher scores for longer genes. When considering only gene models not overlapping known genes, the rate of agreement was significantly lower, particularly for GenScan. This suggests that a combined method accepting input from ab initio and FEAST-like sensors might have a lower error rate by virtue of combining additional sources of information.

Finally, we applied the FEAST algorithms to qualify experimentally derived gene structures, as represented in the University of California Santa Cruz (UCSC) Genome Browser [20] by the rnaCluster and mgcGenes tracks. These respectively denote gene boundaries deduced from clustering spliced ESTs and mRNAs against the genome and cDNA clones from the Mammalian Gene Collection [21]. We again found that longer gene structures are typically recognized at higher levels of certainty by FEAST (Figure 3, center and bottom left). Importantly, the success rate was only moderately lower when excluding gene structures overlapping those in the knownGenes track on which the FEAST method was trained; e.g., for 69% of rnaCluster entries not corresponding to known genes, the orientation was properly predicted by FEAST, with only 4.3% being mispredicted.

### Whole-Genome Transcript Prediction

We implemented a variation on the maximal segment analysis [18,19] to translate genome-wide FEAST scores into specific transcript predictions. When using a Z score cutoff of 2, this analysis yielded a set of 13,623 human genomic regions predicted to be transcribed, with a median size of 81 kb and covering 1,521.3 Mb of genomic sequence. Using a more stringent cutoff (Z = 3) FEAST identifies 6,579 regions with a median size of 138 kb and encompassing 1,132.1 Mb. In contrast, the training set of known transcripts included 19,449 regions totaling just 1,078.4 Mb. This suggests that the current implementation of FEAST yields predictions that (1) miss short genes (Figure 2), (2) correspond to known genes but extend further than annotated, (3) merge separate genes into combined predictions, and/or (4) reveal novel large transcribed regions.

#### Extensions.

**Figure 4:**
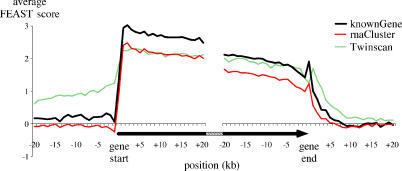
FEAST Scores at Gene Boundaries The average FEAST scores for known genes (thick black, *n* = 10,023), aligned at the position of gene start, show a sharp shift from nonsignificant values (near 0) outside the gene, to significant values at the 5′ end of the gene. The opposite shift is seen at the gene end, although it is more gradual. RNA cluster sequences (thin red, *n* = 13,749) show a very similar graph. Twinscan predictions (dashed green, *n* = 9,131) display positive FEAST scores outside the predicted regions, suggesting an underprediction of gene ends, particularly toward the 5′ end. Known genes, RNA clusters, and Twinscan predictions shorter than 20 kb were excluded from this analysis.

We aligned the known genes at their start and end positions and studied the average FEAST values surrounding gene boundaries. As expected, the observed scores outside the transcripts are low while those within transcripts are significant (Figure 4). The transition at the beginning of genes is much sharper than that observed at their ends, consistent with well-defined transcription start sites and less sharply defined termination signals. We also observed an apparent linear decay in scores from gene start to gene end. The same pattern is obtained when studying expressed mRNAs and ESTs (rnaCluster track). A related but different pattern is observed when analyzing the boundaries of gene predictions, e.g., by Twinscan. According to our analysis, transcripts are expected to extend further than predicted, particularly toward the 5′ end. This is as expected since Twinscan predicts the protein-coding section of genes, to the exclusion of long initial introns and noncoding first exons.

#### Transcription between consecutive genes.

We identified 9,237 intergenic segments between consecutive genes on the same strand (“head to tail”) and calculated integrated FEAST values for them. As a general rule, location between two similarly oriented genes appears to be strongly predictive of intergenic transcription on the same strand, e.g., 52% for intergenic segments in the 10-to 100-kb range, versus 28% for the opposite strand (Figure S5). Such scores frequently cause the bridging of the consecutive genes into a joint prediction, which in some cases might reflect the existence of biologically functional “chimeric” transcripts.

#### Novel transcribed regions.

We identified 5,286 regions that are predicted to be transcribed (Z score ≥ 2) but do not overlap with any previously known genes on the same strand (Figure S6). In this case, “known genes” collates annotated genes and predictions from the knownGene and the ensEmbl tracks in the UCSC database. While the median length of the FEAST predictions is approximately 50 kb, many are less than 20 kb long. A more stringent cutoff (Z ≥ 3) yields 1,293 predictions, with median size of approximately 130 kb. Some of the FEAST predictions may encompass more than one gene each, and we expect many of the predictions with Z scores between 2 and 3 to represent novel genes. Our analysis of the whole human genome suggests the potential existence of thousands of heretofore-unidentified genes.

Several of the new predictions have been confirmed by gene annotations that were introduced at a later stage, i.e., they did not contribute to the training set. For example, a 112-kb-long prediction in 19q13.42 (Z = 8.8) corresponds to a recently published microRNA (miRNA) cluster [22]. Our FEAST analysis suggests that this miRNA cluster may be polycistronic, transcribed as a unit from the CpG island near the 5′ end of the FEAST prediction (Figure S7).

Since many computational gene prediction strategies start by masking the sequence for repeats, we tested whether the novel FEAST predictions are substantially enriched in repetitive sequences when compared to previously known genes. We found this not to be the case: the repeat content of known genes and that of novel FEAST predictions are comparable (Figure S8).

Finally, the possibility exists that some of the novel predictions correspond to genomic regions that are transcribed for miscellaneous reasons other than gene encoding, e.g., chromatin remodeling [23].

### Comparison to Genome Tiling Experiments

With the availability of experimental expression data from dense genomic tiling microarray experiments for ten human chromosomes [2], we asked how successful the FEAST predictions are at identifying novel transcribed regions not already included within known genes. By collating 2,162,170 transcribed fragments (“transfrags”) from 11 experiments, we created a nonredundant set of 503,650 transfrags, of which 260,628 are already included within known genes. FEAST predictions with Z > 3 encompass 76,649 additional transfrags, i.e., 31.5% of those previously unaccounted for by known genes (Table S1). Taking into account that the novel FEAST predictions span only 25.8% of the chromosomal sequence outside known genes, FEAST predictions are very significantly enriched in novel transfrags (Z > 61, *p* < 10^−15^). A significant enrichment was observed for all the chromosomes except the very highly annotated chr19 and chr22. The strongest enrichment ratios were observed for chrY, chr13, and chr21 (Table S1).

We next considered the possibility that the genomic tiling data may include noise in the form of scattered, spurious transfrags. We therefore clustered the transfrags by joining those separated by less than 1 kb of unique sequence (i.e., excluding interspersed repeats) and using maximal linkage. We excluded all resulting clusters less than 5 kb long, which yielded 14,302 clusters including 311,441 transfrags. Ninety percent of the clusters include four to 50 transfrags each, with a typical constituency of ten transfrags per cluster. As for the unfiltered data, the novel FEAST predictions for all chromosomes are significantly enriched in transfrags except for chr19 and chr22. Interestingly, filtering disjoint transfrags from the data set increases the enrichment ratios, particularly for chr7, chr13, chr21, and chrY (Table S1).

Finally, we compared the enrichment ratios observed when partitioning the transfrags by source: polyA+ versus polyA− and cytoplasmic versus nuclear. We observed the highest enrichment ratios for transfrags derived from polyA− and nuclear samples (Table S2). These results suggest that the FEAST algorithms can help identify novel transcribed regions beyond those already annotated as known genes.

### Multidimensional Scaling Analysis

**Figure 5:**
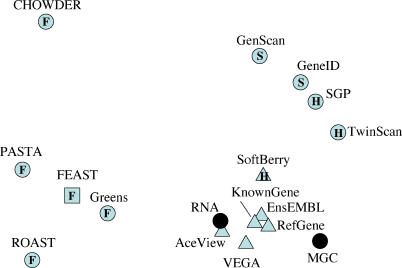
Genomewide Comparison of Gene Annotations The matrix of disagreement measures for all pairs of annotation methods is represented by point in two dimensions using MDS. Filled black circles represent experimentally observed transcripts, the vast majority being in the “RNA” set. Triangles represent methods involving significant manual curation and/or based on the RNA set. “S,” “H,” and “F” represent methods based on gene structure prediction, hybrid methods (gene structure and sequence similarity), and methods measuring footprints of transcription, respectively. The combined FEAST method was excluded from the MDS analysis, and its projected location (squared F) was calculated later (see Materials and Methods). Note that, like geographical maps of intercity distances, MDS representations have no axes.

We used multidimensional scaling (MDS) [24] to compare the genomewide FEAST predictions to those from other gene prediction methods and gene annotations. We devised a distance metric representing the disagreement between the methods, calculated this distance for all possible pairs of methods, and represented these distances by points in two dimensions. The resulting visualization (Figure 5) reveals three main clusters: one including the observed RNA structures and all methods involving curation, a second one including the ab initio and hybrid gene prediction methods, and, finally, a more dispersed cluster including the FEAST components. These results suggest that the FEAST predictions identify a different subset of genes than established gene prediction methods and that a future integrated method incorporating ab initio gene structure prediction, database comparisons and transcriptional side effects could provide a much closer unsupervised approximation to the observed mRNA (transcriptome) data. A similar analysis including randomized versions of the same annotations indicates that the results of FEAST analysis are nevertheless similar in nature to those obtained by other methods (Figure S9).

### Experimental Testing

We performed an initial experimental validation for some of the newly predicted novel transcribed regions.

#### *CPHL1:* A novel ceruloplasmin-related gene.

**Figure 6:**
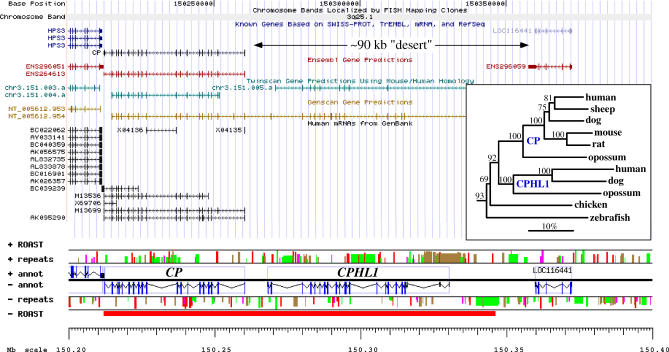
*CPHL1,* a Novel Ceruloplasmin-Like Gene Standard UCSC Genome Browser view of the *CP* locus showing a 90-kb “desert” separating it from the next known gene, *LOC116441,* and GESTALT view of the same locus, indicating the extent of the transcribed region predicted by ROAST (red bar in ROAST track) and the predicted gene structure for *CPHL1.* Interspersed repeats are color-coded, with red, green, pink, and brown bars representing Alu, MIR, LINE, and other repeats, respectively, and bar height indicating repeat age (younger repeats are taller); the megabase scale starts at the p telomere. The newly discovered gene overlaps with a gene structure predicted by Twinscan (chr3.151.005.a) but shares only seven of 21 exons, one imprecisely. GenScan predicts a much longer structure continuous with the *CP* gene, sharing 14 exons with *CPHL1*, of which ten are precisely predicted. Inset: Phylogenetic analysis of the CP/CPHL1 family rooted using the hephaestin protein sequence as outgroup. Numbers above branches represent percentage bootstrap support over 1,000 replicates; the horizontal bar indicates 10% divergence along each branch.

Ceruloplasmin (ferroxidase) is a medically important metalloprotein that evolved by internal tandem triplication [25]. ROAST analysis of the ceruloplasmin gene locus (3q25.1) suggested the presence of a transcript significantly longer than the 50-kb-long *CP* gene [26]. The predicted transcribed region outside *CP* has neither annotated known genes nor mRNA data (Figure 6) and very few spliced EST clones (AA994511, AI217109, BX113166, and BG189564). Using the GESTALT Workbench [27], we identified a putative new gene structure (Figure 6) yielding a predicted protein sequence 57% identical to CP and related to hephaestin (HEPH). This new gene, which we named *CPHL1* (ceruloplasmin and hephaestin-like), is absent from the orthologous locus in mouse and rat genomes, and there is no evidence for its presence elsewhere in those genomes. Orthologs of *CPHL1* exist in the dog and opossum genomes, located immediately upstream of *CP* as in human. Phylogenetic analysis suggests that this probable metal transport protein is a mammalian-specific duplicate of ceruloplasmin that was later lost in rodents (Figure 6, inset). Using PCR primers designed to be specific for the predicted ORF, we observed its expression in prostate cancer CL1 cells [28] and in a mixture of mRNAs from over 30 human tissues. We identified two different isoforms of *CPHL1*, suggesting alternative splicing.

#### *AGBL1:* A novel putative zinc carboxypeptidase.

**Figure 7:**
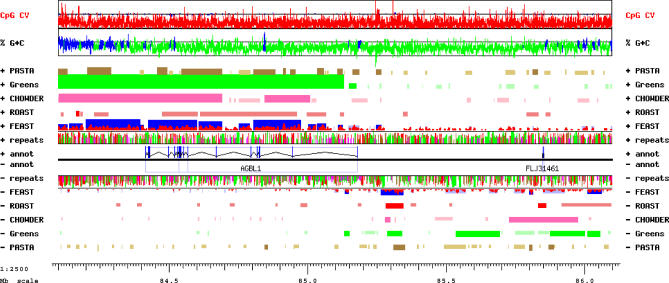
GESTALT View of the *AGBL1* Locus between the *AKAP13* and *NTRK3* Genes on Human Chromosome 15, 84.1 to 86.1 Mb from the p Telomere PASTA, Greens, CHOWDER, and FEAST predictions are displayed for each strand in brown, green, pink, and red, respectively, with lighter shades indicating less significant scores. In the FEAST track, actual scores are indicated in red, and maximal segments are displayed in blue. The *AGBL1* gene structure was modeled based on translated sequence similarity to the AGTPBP1 protein.

FEAST analysis of a 2-Mb “gene desert” between the *AKAP13* and *NTRK3* genes on human chr15 indicated the presence of a transcript spanning at least half of this region. Lacking reliable gene predictions or observed transcripts, we used FASTY [29] to perform a sensitive translated comparison of every nonrepetitive sequence in this locus to protein databases, followed by detailed manual annotation of additional potential exons using the GESTALT Workbench [27]. We predicted a novel gene encompassing 765 kb of genomic sequence and including at least 21 exons (Figure 7). Using specific PCR primers, we observed its expression in the ovarian cancer cell line IGROV-1 [30] and in a mixture of mRNAs from over 30 human tissues (Figure S10). The predicted protein sequence, currently incomplete at its amino end, is at least 940 amino acids long and includes a zinc carboxypeptidase domain (smart00631.10, pfam00246.11). This novel gene, called *AGBL1*, is most closely related to the zinc carboxypeptidase *AGTBPB1*, from which it diverged prior to the teleost-tetrapod split (not shown). In mice, *Agtbpb1* (*Nna1*) is expressed in regenerating motor neurons [31], suggesting that the expression of *AGBL1* may also be developmentally regulated and highly specialized. After our analysis, the sequence of a cDNA clone corresponding to the 3′ half of the predicted structure of *AGBL1* has been deposited in GenBank and annotated as being expressed in prostate tissue.

#### *LOC401237:* A novel large nonprotein-coding gene.

**Figure 8:**
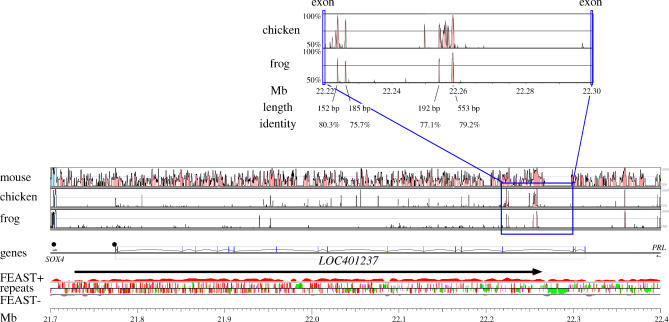
The Highest-Scoring Novel Predicted Transcript, *LOC401237* VISTA and GESTALT analyses of the *LOC401237* locus, showing sequence conservation with the mouse, chicken, and frog orthologous loci; the observed intron-exon structure of *LOC401237* and location of neighboring genes, with black circles representing CpG islands; the integrated FEAST scores for the forward (+) and reverse (−) strands, with the black arrow representing the calculated maximal segment; the repeat distribution on both strands, with red, green, pink, and brown bars, respectively, representing Alu, MIR, LINE, and other repeats, and bar height indicating repeat age (younger repeats are taller); the megabase scale, range 21.7 to 22.4 Mb from the p telomere. Inset on top: Detail on the conserved intronic noncoding sequences, between two nonconserved exons.

The strongest novel FEAST prediction not overlapping any previously known gene indicated the presence of a novel transcript between the *SOX4* and *PRL* genes on human chr6, spanning over 530 kb (Figure 8). Over 30 spliced ESTs and mRNAs from a wide variety of tissues, including embryonic stem cells, adult brain, uterus, renal epithelial cells, and eye lens, have been assigned to this locus. With the single exception of AK126168, all of these transcripts are annotated in the orientation predicted by FEAST and are the basis for the gene model *LOC401237*. We designed PCR primers based on AK026189, which spans most of the predicted region, and experimentally verified its expression in the mRNA panel. No open reading frame can be identified by splicing the observed exons; a translated comparison by FASTY [29] against the protein databases fails to identify any significant similarity. Furthermore, while orthologs for some of the exons are identifiable in other vertebrate genomes (dog, mouse, rat, opossum, chicken, and frog), the exact exon boundaries are not conserved, and many “frameshifting” mutations appear to have been accepted in the evolution of these exons. In fact, the exons do not display higher conservation levels than the introns, which in turn contain several conserved noncoding sequences (CNSs) (Figure 8). The function of the intron CNSs remains to be determined. CNSs also exist between *LOC401237* and *PRL* (Figure 8). Significant FEAST biases extend 320 kb beyond the 3′ end of *LOC401237,* suggesting the presence of additional novel transcripts in this locus.

## Discussion

Current methods for gene prediction perform well on genes that are “typical” in several respects, including number and length of exons, length of introns, quality of splice sites, and conservation (similarity to known genes). Some divergent genes may be difficult to discover by experimental observation of transcripts if their range of expression is restricted to one or a few cell types or if they are expressed at very low levels. It is significantly more difficult to identify and produce correct models of genes with extremely long introns or short exons or that have diverged extensively from other genes; this is particularly true for genes that do not code for proteins. Such genes would be composed almost entirely of intronic sequence and could be practically “invisible” to current computational gene prediction methods.

We found that transcribed sequences hold significant information about the direction of transcription, in the form of significant orientation biases of (1) nucleotide composition, (2) mutations within interspersed repeats, (3) the interspersed repeats themselves, and (4) PASs. We implemented and integrated four algorithms (Figure 1). Greens and CHOWDER rely on biases introduced by transcription in the germline, but the generality of the skews suggests that this includes a large fraction of the genes [32]. ROAST and PASTA reflect functional transcription in both autosomal and germline tissues. The observed skews are evidence for sustained transcription over evolutionary time, and are not caused by “transcriptional noise,” i.e., indiscriminate transcription of random regions of the genome, that complicate the interpretation of most experimentally based transcript identification methods.

For the purpose of the current work, interspersed repeats have two interesting characteristics. First, since copies generally do not adopt a function within the genome, they accumulate substitutions in a neutral fashion. The availability of sufficient copies allows for a relatively accurate reconstruction of the element sequence at the time of integration, while comparison of the extant copies against these “consensus sequences” gives an accurate account of the frequency spectrum of neutral substitutions. These data have, for example, been used to derive log-odds matrices for comparison of interspersed repeats to a consensus database in the program RepeatMasker as well as for the alignment of genomic sequences of different mammals [33,34]. We exploit this aspect of interspersed repeats by measuring the strand-specific substitution biases in repeats (CHOWDER) and the changes in PAS strength (PASTA) to predict the presence and orientation of a transcribed region. Second, while decayed interspersed repeats are generally relatively inert, except for promoting homologous recombination, at the time of integration they contain functional transcription regulatory signals that can affect nearby gene transcription, as exemplified by the discovery of oncogenes constitutively expressed from nearby retroviral LTR [35]. Probably mostly because of transcriptional disruption by their PAS, LINE and LTR elements are underrepresented in the forward orientation of genes [13,14,16,36]. Although the nature of the interaction of other interspersed repeats with genes is less clear, their distribution is nonrandom with respect to the location of genes as well. Notably, the location of lineage specific (and therefore independently accumulated) SINEs in different mammals is remarkably similar [37,38]. Thus, the distribution pattern of repeats harbors significant information concerning the location of genes. We have utilized this aspect to infer transcription unit locations, by quantifying the abundance of each type of repeat in the forward and reverse strand of genes. The data were stratified by GC level, to accommodate the large-scale correlation of repeat densities with isochores.

The “transcriptional footprints” described here have some conceptual similarity to “content” methods like coding potential and coding sequence compositional biases. While those are limited to coding exons, the signal of transcriptional footprints can be observed throughout the length of the transcript, the vast majority of which is usually intronic in nature. Furthermore, while the “content” methods detect deviations from the sequence composition expected under a random model, the FEAST methods detect significant strand biases of selected signals, regardless of their absolute frequency. A generalized linear model for transcript detection was published [39], integrating nucleotide skews and some repeat densities (not strand biases). Sémon and Duret used a 20-kb sliding window approach to identify putatively transcribed regions but found their method to be insufficiently accurate for automatic gene prediction.

The basic model underlying the four FEAST methods assumes the accumulation in introns and UTRs of strand-biased signals arising as side effects of transcription. Several deviations from this model can be postulated: (1) a significant proportion of the genomic region included in a gene might not be transcribed, as is the case for somatic rearranging immune loci; (2) antisense overlapping transcripts may lead to partial signal cancellation; and (3) a gene may have long coding exons, or a large number of exons separated by short introns. Furthermore, the statistical model used assumes independence between the observed signals, which may not be true for (4) arrays of tandemly duplicated sequences or (5) interspersed repeats “homing” into similar repeats, e.g., Alu [40]. Finally, sensitivity may suffer if there is insufficient signal, e.g., for (6) short genes or (7) evolutionarily new transcribed regions, or if the signal was lost by (8) inversions within the introns or other genomic rearrangements. Conversely, (9) decaying pseudogenes derived from genomic duplications may yield spurious signals. Most of these model deviations are expected to lead to false negatives, suggesting that FEAST may be underpredicting the number and/or the extent of genes.

In the current implementation of FEAST, the four algorithms are combined with equal weights, except for Greens and CHOWDER being weighted according to the repeat fraction. This will be improved in future versions by identifying context-dependent optimal weights for the different algorithms. Since the mutation-based methods refer to germline expression but the selection-based methods reflect functional importance at any developmental stage, additional functional information could be obtained by using different combinations of the four algorithms. Finally, a promising area for future development is the joint gene prediction on orthologous regions, by collating biases accrued independently in different species lineages.

**Figure 9:**
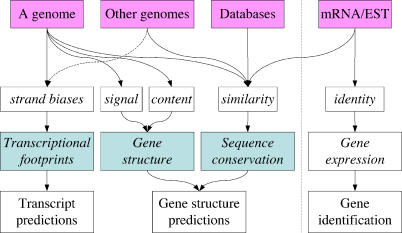
A Third Basic Concept By studying various sources of sequence information (pink boxes), genes have been identified using a variety of computational methods based on the identification of gene structure and/or the identification of sequence conservation. The FEAST methods represent a third basic concept, in which sustained transcriptional activity is inferred by its mutational and selective effects on the genomic sequence, the “transcriptional footprints.” Light blue boxes indicate the three basic concepts for gene prediction. The dashed vertical line separates gene prediction (to the left), from gene identification (to the right): the latter is based on the analysis of sequences expressed from the same locus.

Sequence-based gene prediction has long been dominated by methods based on modeling gene structure and sequence comparisons, followed by extensive expert curation. It might have appeared impossible to detect genes from genomic sequence without identifying splicing signals or sequence conservation and not even relying on the genomic localization of experimentally observed expressed sequences. We presented here a third basic concept (Figure 9), i.e., the genomic effects of sustained transcription, and four transcript prediction algorithms based on it. It is important to stress that the methods described here differ from conventional gene prediction methods in that they do not lead to detailed prediction of the intron-exon structure of the predicted genes but rather identify the overall extent and orientation of the transcribed regions. To achieve a complete gene model, further analyses are required.

In addition to yielding hypotheses for correcting 5′ incomplete gene annotations and novel independent predictions, many of which cannot be detected by gene structure or similarity, the new algorithms are complementary to existing methods (Figure 5). We therefore expect these tools to add valuable information when integrated with the algorithms based on gene structure and sequence similarity, as a further step toward achieving the sensitivity and specificity required for fully automated whole-genome annotation.

## Materials and Methods

### Overview of computational analyses.

We analyzed the human genomic sequence [12]. The whole genome was analyzed using RepeatMasker (http://repeatmasker.org), Tandem Repeats Finder [41], polyadq [17], and custom Perl scripts to calculate nucleotide compositions and enumerate mutation events within interspersed repeats based on RepeatMasker alignment files. We identified transcribed genomic regions based on the annotation of “known genes,” suppressing those annotated as overlapping other genes. We trained on this data set by counting frequencies of the observed elements (repeats, G + T nucleotides, mutations within repeats, PASs) in both orientations and within nonoverlapping 1-kb bins and then calculated log-likelihood ratio parameters for each, with appropriate stratifications. Using these empirically derived parameters, we reanalyzed the known genes to assign a score to each. We further analyzed the entire human genome to predict transcripts, using a maximal segment analysis approach [18,19] with separate extension models for transcribed and intergenic sequence. Finally, we compared the overall distribution of FEAST predictions to those obtained by several gene prediction programs and mRNA/EST annotations.

### Definition of training sets.

We obtained the July 2003 freeze of the human genome (hg16, based on NCBI Build 34) and its annotation database from the UCSC Genome Bioinformatics Site [20]. For training the algorithms, we defined two data sets: (1) the “TRON” set of transcribed sequences (which are mostly inTRONs), reverse-complemented if needed, such that all genes are transcribed in the same (forward) strand, and (2) the “TERG” set of untranscribed (inTERGenic) sequences, including all sequences not annotated to be transcribed, but excluding any gaps longer than 1 kb (e.g., centromeres). We based our definition on the “knownGene” track of the UCSC Genome Browser [20], which collates annotation about protein-coding genes based on proteins from SWISS-PROT, TrEMBL, and TrEMBL-NEW, and their corresponding mRNAs from GenBank, and includes 38,482 entries totaling 1,860.8 Mb. We removed redundancy (e.g., alternative transcripts of the same gene) by maximal clustering of overlapping features on the same strand, yielding 18,956 clusters spanning 1,023.9 Mb. We observed very limited overlap of transcripts on opposite strands (totaling 15.4 Mb). We excluded from the training set all transcript clusters overlapping known transcripts on the opposite strand. We performed a similar analysis on the mouse genome [37] (October 2003 freeze, mm4, based on NCBI Build 32).

We stratified the genomes by G + C content into five nearly equal parts by sequence length. Any genomic sequence (and hence the repeats in it) is classified as having low, medium low, medium, medium high, or high G + C content. The optimal cutoffs to obtain such separation are calculated to be 35.9%, 38.5%, 41.3%, and 45.4% G + C for the human genome and 38.0%, 40.0%, 42.3%, and 45.2% G + C for the mouse. When considering sequences in the TRON sets, the forward strand was defined to be the one running 5′ to 3′ in the direction of transcription of the gene. For intergenic sequences (TERG set), both strands are equivalent, and the forward strand is arbitrarily defined to be that which runs from the p telomere to the q telomere of the chromosome.

### Genomewide repeat analysis.

We identified and classified all SINEs, LINEs, LTR, and DNA elements in the human and mouse genomes, using the standard classification implemented in the RepeatMasker software (version of 23 June 2003, at sensitive settings; http://repeatmasker.org). We tabulated the number of repeats in each of the genomic sets (Table S1), excluding simple sequence repeats and low complexity regions, and calculated their density as repeat counts per Mb of sequence. RepeatMasker identifies which repeat fragments likely derive from one single original repeat, avoiding most “double-counting” of disrupted repeat elements.

We stratified the repeats by G + C content as described above. We further stratified the repeats by age. The percentage of divergence of each repeat from its consensus sequence was used as an estimate of its age, assuming that an older repeat would have diverged more from its corresponding family consensus than a younger one: we subdivided repeats into nonoverlapping 5% divergence bins.

This analysis resulted in a detailed catalog of repeat counts, stratified by repeat type, repeat family, G + C content, divergence level, and orientation (see below). For example, in the human genome we observed 17.55 LINE/L1 repeats less than 5% divergent from consensus, per Mb of low G + C content sequence in intergenic regions, but 14.86 such repeats per Mb in similar regions that are annotated to be transcribed. The data sets are available at http://repeatmasker.org/FEAST.

### Greens: Quantification of G + T skews.

We implemented a variation on the nucleotide composition method published by Green et al. [8]. Instead of using the predicted equilibrium frequency of 0.527, we studied transcribed, nonrepetitive sequences in 100-bp bins and plotted the distribution of observed log-likelihood ratios as a function of the G + T skew (Figure S1B). This curve shows a clear saturation effect for extreme G + T skews, which are not modeled using a single log-likelihood ratio score. To avoid artifacts of low sampling, we fitted an arbitrary function of the shape:


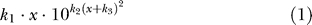


where *x* is the G + T skew. Best fit was obtained with *k*_1_ = 3.2, *k*_2_ = −2, and *k*_3_ = 0.07 (red fit curve in Figure S1B). The relationship between the forward and reverse log-likelihood ratio curves is given by:


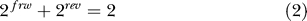


When studying a genomic sequence for prediction purposes, the Greens score of a 1-kb bin was calculated as the sum of the scores of 100-bp nonrepetitive windows contained within it and normalized to Z scores in the same way as for ROAST scores (described below).

### CHOWDER: Quantification of mutations within repeats.

We studied the alignment files produced by RepeatMasker to identify all the single-nucleotide differences between each interspersed repeat (with the same exclusions as in ROAST) and its corresponding consensus sequence. These differences can be assumed to represent directional mutations from the consensus sequence state to the extant sequence state (Figure S2A). This method is akin to the “substitution rate” method described by Green et al. but is applied to the content of interspersed repeats. Since most repeats cannot be assumed to have approached mutational equilibrium, the method used here is empirical. We excluded from the analysis CpG mutations and tabulated only isolated mutations, defined as those for which the 5′ and 3′ neighboring sites were identical between the repeat consensus and the extant sequence. We defined the orientation of a mutation as that in which a purine has mutated. For example, a mutation changing a forward-strand C in the consensus sequence to a T in the extant sequence is considered instead a change from a reverse-strand G into an A. Thus, instead of 12 possible mutations, we considered six possible mutations, each with two possible orientations. We tabulated their frequencies relative to the orientation of transcripts and found them to be biased (Figure S2B). We then calculated log-likelihood ratios and integrated the observed values in the same way as for ROAST scores (described below).

### ROAST: Quantification of repeat orientation biases.

Interspersed repeat consensus sequences in the RepeatMasker/RepBase Update databases are oriented in the direction of transcription of the transposable element, which is usually recognized by the coding region. This orientation was unknown for a fraction of repeats, primarily LTR sequences without an associated internal sequence or noncoding DNA transposons. We determined orientations for these based on similarity to oriented elements or discovery of internal sequences or coding region in extended consensus sequences, leaving for the human genome only the Mariner-like MADE and MER1-group MER119 DNA transposons nonoriented and excluded from further analysis. A significant fraction of L1 repeats show an inversion, attributed to a “twin priming” mechanism [42]. We defined the orientation of such inverted L1 repeats as the orientation of the segment corresponding to the 3′ end of the L1 consensus sequence. Failure to implement this correction can lead to the underestimation of L1 bias in transcripts and to the artifactual observation of significant L1 orientation biases in intergenic sequences.

When a repeat is located within a transcript, we refer to it as a “forward repeat” if its orientation is the same as that of the transcript; otherwise, we consider it a “reverse repeat.” For each repeat family, stratified by repeat age and regional G + C content, we calculated a score based on log-likelihood ratios, with the null hypothesis claiming the expectation of observing equal numbers of forward and reverse repeats. If F and R represent the observed number of repeats in the forward or reverse orientation relative to the enclosing transcript, the log-likelihood ratio contribution to the ROAST prediction of transcription in the same orientation as such a repeat is given by:


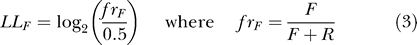


For example, we observed 26,191 mid-aged reverse Alu repeats within transcripts of intermediate G + C content but only 19,976 in the forward strand. Therefore, as only 43.27% were observed in the forward strand (and not the expected 50%), the score contribution for a transcription claim in the same orientation as such an Alu repeat is log_2_(0.4327/0.5) = −0.209, while the contribution to the claim of transcription in the reverse orientation receives a score of log_2_[(1 − 0.4327)/0.5] = 0.182. The −0.209 figure for the forward strand is different from the value plotted in Figure S3B, because the latter includes information about all Alu repeats in the genome.

For testing genomic sequences, we subdivided them into nonoverlapping 1-kb bins and assigned to each bin those interspersed repeats for which the midpoint lies within the bin. The ROAST score for a bin is the sum of the log-likelihood scores of its repeats, normalized to the average and standard deviation of the distribution of scores obtained if the same repeats had been observed in random orientations. Therefore, the ROAST score is expressed as a Z score, or standard deviations away from the mean value (under the null hypothesis), i.e.:


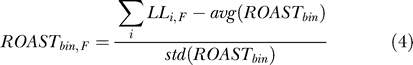


with:


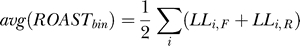


and


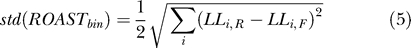


where *i* represents each repeat observed in the genomic bin studied, and *LL_i,F_* is either *LL_F_* or *LL_R_* according to whether repeat *i* is in the same or opposite direction as the direction of transcription being tested.

### PASTA: Quantification of PASs.

Based on the RepeatMasker alignment files, we produced a “sequence mask,” which is a version of the genomic sequence in which each interspersed repeat is replaced by the corresponding segment from its consensus sequence (Figure S4A). We then predicted PASs on both the unmodified genomic sequence and the “sequence mask” using polyadq [17] with default parameters. We tabulated the frequencies of PAS relative to the orientation of transcripts, stratifying them by T/A skew [i.e., T/(T + A)], PAS consensus (AATAAA or ATTAAA), and PAS strength, and subtracting PAS observed within repeats in the “sequence mask.” We then calculated log-likelihood ratios as for ROAST, but instead of the expected value of 0.5, we used the ratio observed in intergenic sequences of similar T/A skew; this is to compensate for the expected depletion of forward-strand PAS in T-rich sequences (Figure S4B).

### Integration of scores.

The FEAST score for a single bin is calculated from the individual method scores as follows:


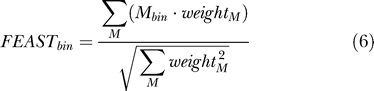


where *M* is each of ROAST, Greens, CHOWDER, and PASTA, and *weight_M_* is the relative weight given to each method. Currently, the weight of Greens' method is set to the fraction of unique sequence in the sequence bin, the weight of CHOWDER is set to the fraction of repetitive sequence, and the weight of the other methods is set to 1. It is therefore possible to calculate a FEAST score for any combination of methods.

The FEAST score of the range of bins *x..y* (inclusive) is calculated as:


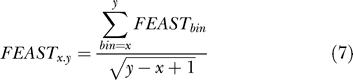


We identified maximal scoring segments [18,19] by linearly extending segments with positive scores and reporting only those segments with combined score FEAST_x..y_ > 2 in either orientation. To avoid merging distinct high-scoring segments separated by a region of negative or low scores, we tabulated the frequency of low-scoring segments within annotated genes, and we used these values to determine the probability that a low-scoring segment following a maximal scoring segment is still included within the same predicted transcript. Where this probability was less than 10^−3^ for 1,000 shuffles, the low-scoring segment was considered to represent the beginning of an intergenic region and was used as the boundary for the previous maximal segment.

### Comparison to genome tiling data.

We obtained the transcribed fragment (“transfrag”) data from the UCSC Genome Bioinformatics Site [20] in 11 files listing transfrags expressed in different experiments [2]. We excluded a very small number of transfrags that were annotated as deriving from chr12, and those from a chr6 haplotype. This yielded a redundant set of 2,162,170 transfrags, which we collated into a nonredundant set of 503,650 transfrags that were expressed in at least one experiment. For each pair of consecutive transfrags along a chromosome, we removed the interspersed repeats from the sequence between the transfrags, and if the remaining sequence was less than 1 kb (an arbitrary cutoff based on the observed distribution of lengths), we considered the transfrags to be linked into a cluster. We then excluded clusters spanning less than 5 kb of (unmasked) genomic sequence. We studied all the transfrag data combined or stratified by sample type (polyA+, polyA−, cytosolic, and nuclear).

### MDS analysis.

We computed for each method M its total extent TE_M_ as the sum of the lengths of all the gene ranges annotated by it (collapsing overlapping ranges on each strand). For each pair of prediction methods x and y (e.g., ROAST and GenScan), we computed the measure of minimal possible disagreement minD_x,y_ as abs(TE_x_ − TE_y_); i.e., the least disagreement will be observed when one set of predictions is entirely contained in the other, and their extents are exactly the same. We then calculated the maximal possible disagreement maxD_x,y_ as TE_x_ + TE_y_ (i.e., maximal when they are totally disjoint), or 2 • length(chrom) − (TE_x_ + TE_y_) when TE_x_ + TE_y_ > length(chrom). The actual observed nucleotide overlap disagreement between the methods obsD_x,y_ is then normalized linearly to the range minD_x,y_..maxD_x,y_, yielding a distance measure equal to 0 when the methods yield identical results and equal to 1 when they are maximally disjoint.

We then created a two-dimensional visualization of the relationships among the methods from the matrix of pairwise distance measures, using the MDS algorithm ALSCAL as implemented by the SPSS statistical system, while specifying a ratio level of measurement with Euclidean distance, thereby creating a metric scaling solution. The technique of MDS seeks to create a configuration of points in two-dimensional space such that the pairwise Euclidean distances between pairs of points are closest to their respective actual distance measures. The ALSCAL algorithm iteratively seeks to minimize Young's S-stress formula 1, which is defined as the square root of the ratio (sum over all pairs of the squared difference between squared actual and squared Euclidean distances) divided by (sum over all pairs of the fourth power of the scaled Euclidean distance). Further details may be found, for example, in Davidson [43] or in Young [44].

In order to avoid giving additional weight to the FEAST components in Figure 4, an initial MDS was obtained with the FEAST components (ROAST, CHOWDER, Greens, and PASTA) but without FEAST itself. The optimal location for FEAST, as displayed, was then computed by minimizing the total S-stress but without changing the locations of the other points. An MDS without excluding FEAST gave substantially similar results.

### Experimental verification.

Selected novel gene predictions were confirmed by PCR amplification from double-stranded cDNA, which was prepared from a mixture of over 30 different human tissues. PCR primers were specifically designed for each gene based on predicted exon sequences. Amplification products were sequenced to confirm their identity and to establish intron-exon boundaries.

Primers were designed using the primer3 software [45]. To achieve higher specificity, primers were designed to have a T_m_ of approximately 70 °C in a two-step cycling program with a 68 °C annealing/extension step. PCR was performed with BD Advantage2 Polymerase Mix (catalog No. 639201; BD Biosciences Clontech, Palo Alto, California, United States) and Human Universal QUICK-Clone II cDNA as template (catalog No. 637260; BD Biosciences Clontech). Universal QUICK-Clone II is double-stranded cDNA reverse-transcribed from RNA prepared from a mixture of over 30 different human tissues (adrenal gland, aorta, bone marrow, brain, cerebellum, cerebral cortex, hyppocampus, thalamus, fat cell, fetal brain, fetal heart, fetal kidney, fetal lung, fetal liver, heart, kidney, leukocytes, liver, lung, lymph node, mammary gland, ovary, pancreas, pituitary gland, placenta, prostate, retina, salivary gland, skeletal muscle, small intestine, spinal cord, spleen, stomach, testis, thymus, thyroid gland, and uterus). Double-stranded cDNA was synthesized from polyA+ RNA using an oligo(dT) primer, purified to remove interfering RNA and genomic DNA, and size-selected to remove fragments smaller than approximately 400 bp. BD Advantage2 DNA polymerase contains built-in hot-start PCR from BD TaqStart Antibody. It is claimed to give consistent and efficient amplifications of up to 18-kb PCR products for a noncomplex template or up to 6 kb for high-complexity genomic DNA. It is also claimed to exhibit an error rate of 25 errors per 100,000 bp after 25 PCR cycles.

The PCR cycle parameters used were optimized for target size 5 to 9 kb as recommended by the manufacturer—melting: 95 °C for 1 min; 35 cycles annealing and extension: 95 °C for 30 s, 68 °C for 6 min; hold: 68 °C for 10 min, 4 °C indefinitely.

The PCR products were visualized under long-wave UV light on 1.2% agarose gel loaded with 1-kb DNA ladder, stained with ethidium bromide. The PCR product bands were cut from the gel and purified by Qiaquick gel extraction kit (Qiagen, Valencia, California, United States). The DNA samples eluted from gel extraction columns were used directly for sequencing reactions or further amplified by PCR prior to sequencing.

Sequencing reactions were performed using 1/16 dilution of Applied Biosystems Big Dye Terminator v3.1 Reaction Mix (Foster City, California, United States). Reactions are performed in 50 cycles on MJ Research PTC-225 thermocycler tetrads (Waltham, Massachusetts, United States) and precipitated with isopropanol and centrifugation. The sequencing ladders were resolved using Applied Biosystems 3730XL sequencer and accompanying base-calling and data quality analyses software.

## Supporting Information

Figure S1 The Corrected Greens Algorithm (A) Schematic describing how excessive G + T skews may not be predictive of transcription.
(B) Log-likelihood ratio contribution of different strengths of G + T skew, within known genes. Skews range from −1 (only A + C) to +1 (only G + T). Observed values are given in blue, and arbitrary fit curve, in red. Highly skewed G + T compositions are observed to be less indicative of transcription than more moderate skews.
(49 KB PPT)

Figure S2 CHOWDER: Transcript Prediction Based on Substitutions within Repeats (A) Schematic describing the integration of a transposable element, potentially truncated, followed by accumulation of generally neutral substitutions. A comparison of the consensus sequence for many copies of this element, approximating the original sequence (filled), to the extant sequence (striped) yields a list of substitutions. To avoid distortions from alignment artifacts, only substitutions flanked by unchanged nucleotides are considered (thick vertical lines). Mutations involving CpG dinucleotides are also excluded.
(B) The chart indicates the total number (in millions) of directional mutational events observed in interspersed repeats within genes in the entire human genome, from repeat consensus to extant sequence. For each mutation, the upper value represents the mutation in the forward strand of the enclosing gene, while the lower value represents the same mutation in the reverse strand. For example, we observed 4.58 × 10^6^ mutations from an A in the repeat consensus to a G in the forward strand of the extant sequence but only 4.11 × 10^6^ mutations from a reverse-strand A in the repeat consensus to a reverse-strand G in the extant sequence (i.e., a T→C mutation in the forward strand). Bold arrows indicate mutations that are more frequent in the forward strand than in the reverse strand.
(29 KB PPT)

Figure S3 ROAST: Biased Repeats (A) Schematic describing the hypothesis of how the introduction of an interrupting signal (red) tends to be rejected, while the same signal in the opposite strand is not disruptive (white) and therefore is neutral. This process yields a strand bias.
(B) The log-likelihood ratio contribution, of a single repeat, to the claim of transcription in the same orientation as the repeat. Negative log-likelihood ratio values represent the prevalence of repeats in the reverse strand. The values for the various interspersed repeat families in human and mouse are correlated. The correlation was calculated based on repeat families that are significantly biased in both lineages, represented by filled icons. SINEs, LINEs, LTR elements, and DNA repeats are shown in red, green, blue, and black, respectively.
(29 KB PPT)

Figure S4 PASTA: PASs (A) A statistical analysis of PASs in transcribed nonrepetitive sequence revealed significant orientation biases, after correcting for nucleotide composition skews. In this schematic, PAS are represented by octagons, with color indicating signal strength (darker represents stronger signals). Within repeats, we found biases in PAS strength changes from the repeat consensus (filled repeat icons) to the extant sequence (open repeat icons).
(B) Expected PAS frequency skew as a function of nucleotide composition skew. Sequences enriched in T in the forward strand are expected to have more PASs in the reverse strand. Since the AATAAA signal is more biased than ATTAAA, its expected random skew is stronger.
(31 KB PPT)

Figure S5 FEAST Scores in Intergenic Segments We selected the regions between consecutive genes in the same orientation and normalized their orientation to the forward strand. These regions show a prevalence of positive FEAST scores, indicating preference for transcription in the same strand as that of the flanking genes. Legend is the same as for Figure 3.
(30 KB PPT)

Figure S6 Novel Gene Predictions, Not Overlapping Any Known Gene or Ensembl Prediction on the Same Strand FEAST score versus prediction length given in kilobases, logarithmic scale.
(109 KB PPT)

Figure S7 GESTALT Workbench Analysis of the miRNA Clusters on chr19:58835001–59000000 From top to bottom: CpG contrast values, %G + C; predictions by PASTA, Greens, CHOWDER, and ROAST on the top strand, and their integration into FEAST; interspersed repeats color-coded by family; gene annotations; annotations and predictions on the bottom strand; Mb scale from the p telomere of chr19. The novel 43 miRNA cluster appears to be transcribed as a unit from the CpG island at 58.84 Mb. The FEAST prediction on the top strand (score = 8.8) is consistent with the orientation of the miRNA genes. The smaller miRNA cluster (including mir-371, mir-372, and mir-373) appears to be transcribed separately.
(39 KB PPT)

Figure S8 Repeat Content of Known Genes and Novel FEAST Predictions There is a large set of annotated known genes (blue) composed entirely of repetitive sequence. The novel FEAST predictions (Z > 3, red) with greater than 90% repeats are mostly satellite-rich pericentromeric regions and most probably represent false positives.
(190 KB PPT)

Figure S9 Expanded MDS Analysis We include here the genomewide annotation of known genes (KG), Ensembl genes (ENS), Twinscan (TW), GenScan (GS), Softberry genes (SB), EC genes (EC), GeneID (GID), RNAs (RNA), Mammalian Gene Collection (MGC), pseudogenes (PS), Exoniphy exons (EX), Exoniphy exons bridged when in the same orientation and within 25 kb of each other, and not separated by exons in the opposite strand (EXB), all combinations of FEAST methods (e.g., CP is CHOWDER and PASTA; RCG includes ROAST, CHOWDER, and Greens), and a randomized version of each annotation method (names appended with “.s” for “shuffled”). The randomized versions are distributed along a wide arc that includes the pseudogenes and are clearly distinct from the unshuffled annotations (including the four FEAST methods and all their combinations). Note that, like geographical maps of intercity distances, MDS representations have no axes.
(105 KB PPT)

Figure S10 Experimental Verification of Expression of *AGBL1*
(A) Lane 3: Two PCR product bands amplified using *AGBL1*-specific primers were cut (indicated by the white arrows) under long-wave UV light on agarose gel stained with ethidium bromide. Lane M: 1-kb DNA ladder (Invitrogen, Carlsbad, California, United States). Lane 1: 335-bp human actin gene PCR product as positive control. The PCR products in gel slices were purified with Qiaquick gel extraction kit (Qiagen).
(B) The DNA products purified from the lower band (~750 bp) and higher band (~1,350 bp) were further amplified by PCR before sequencing. Lane M: 1-kb DNA ladder. Lanes 2 through 4: PCR products amplified from *AGBL1* lower band at 55, 62, and 68 °C PCR annealing temperature, respectively. Lanes 6 through 8: PCR products amplified from *AGBL1* higher band at 55, 62, and 68 °C PCR annealing temperature, respectively.
(482 KB PDF)

Table S1 Comparison to Affymetrix Transfrags (A) All transfrags in the ten chromosomes listed.
(B) The filtered transfrags after clustering. All FEAST predictions have Z > 3. The columns indicate the chromosome; the total number of transfrags; the number of transfrags in known genes, in FEAST predictions, in FEAST predictions but outside known genes, and the percentage of transfrags outside known genes that were included in FEAST predictions; the effective chromosome length (excluding gaps); the length of sequence included in known genes, in FEAST predictions but outside known genes, and the percentage of sequence outside known genes that was included in FEAST predictions; and the novel/out ratio between number of transfrags and sequence length (enrichment), its standard error, its Z score, and the probability to observe such enrichment under the null hypothesis. Numbers shown in italics are not significant (*p* > 0.01).
(23 KB XLS)

Table S2 Enrichment Ratios for Stratified Transfrags For each chromosome, and for all chromosomes in combination, we calculated enrichment ratios as in Table S1, stratifying by sample type. Numbers shown in italics are not significant (*p* > 0.01).
(17 KB XLS)

### Accession Numbers

The GenBank (http://www.ncbi.nlm.nih.gov/Genbank) accession numbers for the genes and gene products discussed in this paper are AA994511, AI217109, BG189564, BX113166, *CP* (NT_005612.954), hephaestin protein sequence (NP_620074.1), gene model *LOC401237* (XM_379398, AK056872, and AK026189), and NT_001520.

## Abbreviations

CHOWDER: CHanges Oriented Within DispErsed Repeats
CNS: conserved noncoding sequence
FEAST: fast empirical algorithms suggesting transcripts
MDS: multidimensional scaling
miRNA: microRNA
PAS: polyadenylation signal
PASTA: PAS transcript analysis
ROAST: repeat orientation analysis suggesting transcripts
UCSC: University of California Santa Cruz
