# Supplementary material for: A Third Approach to Gene Prediction Suggests Thousands of Additional Human Transcribed Regions
Source: PLoS Comput Biol. 2006 Mar 17;2(3):e18. doi: 10.1371/journal.pcbi.0020018 (PMC1391917; doi:10.1371/journal.pcbi.0020018)
Supplement: Supplementary file 1 [file pcbi.0020018.sg010.pdf]

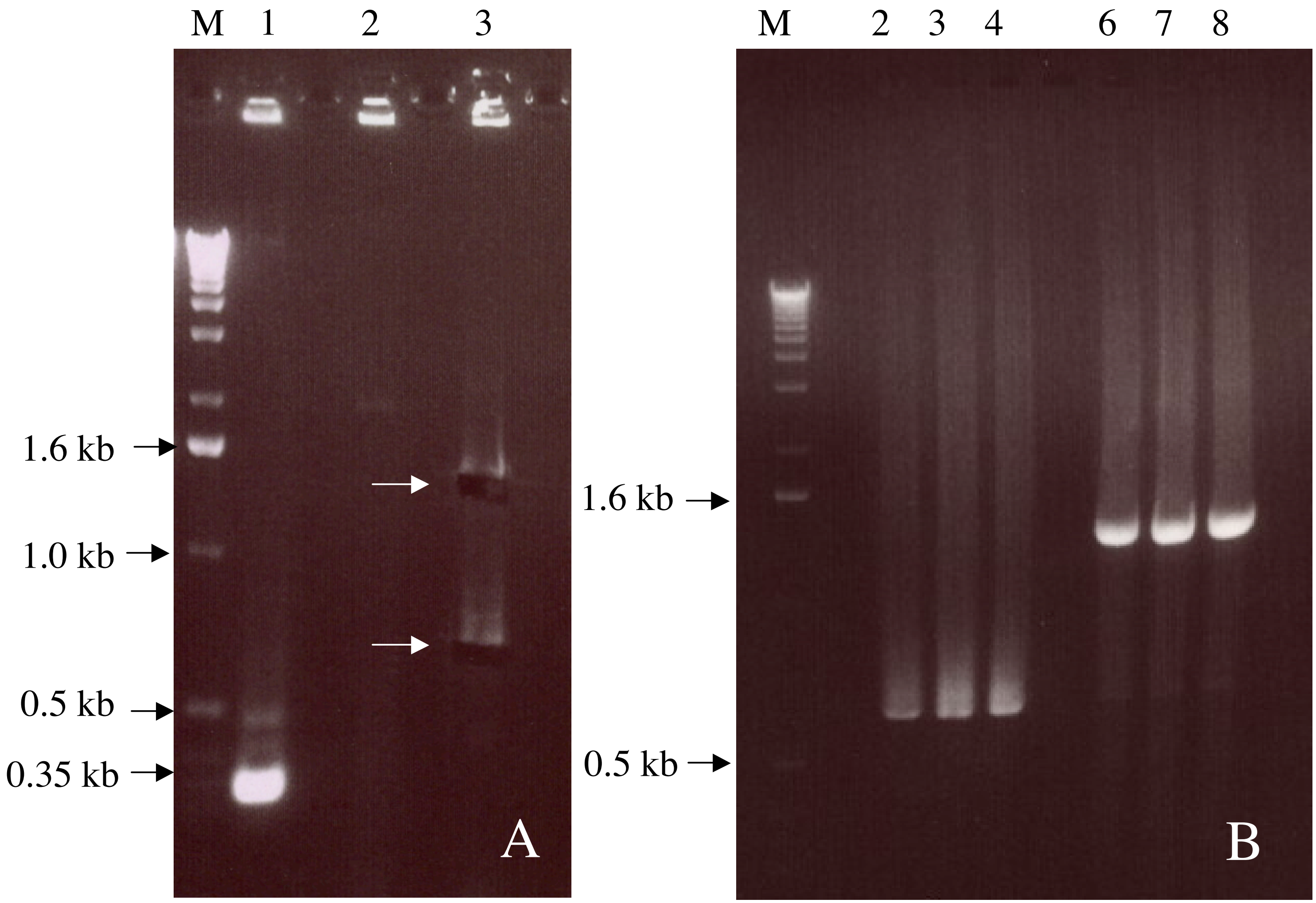

Figure S10
